# Concomitant pulmonary disease is common among patients with extrapulmonary TB

**DOI:** 10.5588/ijtld.21.0501

**Published:** 2022-04-01

**Authors:** S. V. B. Y. Shivakumar, C. Padmapriyadarsini, A. Chavan, M. Paradkar, B. M. Shrinivasa, A. Gupte, K. Dhanasekaran, B. Thomas, N. Suryavanshi, C. K. Dolla, S. Selvaraju, A. Kinikar, S. Gaikwad, R. Kohli, G. N. Sivaramakrishnan, N. Pradhan, L. E. Hanna, V. Kulkarni, A. DeLuca, S. R. Cox, L. Murali, K. Thiruvengadam, S. Raskar, G. Ramachandran, J. E. Golub, N. Gupte, V. Mave, S. Swaminathan, A. Gupta, R. C. Bollinger, for the CTRIUMPh-RePORT India Study Team

**Affiliations:** 1 Johns Hopkins India, Pune, India; 2 Indian Council of Medical Research-National Institute for Research in Tuberculosis, Chennai, India; 3 Byramjee Jeejeebhoy Government Medical College-Johns Hopkins University Clinical Research Site, Pune, India; 4 Johns Hopkins School of Medicine, Baltimore, MD, USA; 5 Byramjee Jeejeebhoy Government Medical College, Pune, India; 6 District Tuberculosis Office, Thiruvallur, India; 7 Johns Hopkins Bloomberg School of Public Health, Baltimore, MD, USA; 8 World Health Organization, Geneva, Switzerland

**Keywords:** *Mycobacterium tuberculosis* infection, infectious disease transmission, retraining, symptom evaluation, cough

## Abstract

**BACKGROUND:**

Microbiologic screening of extrapulmonary TB (EPTB) patients could inform recommendations for aerosol precautions and close contact prophylaxis. However, this is currently not routinely recommended in India. Therefore, we estimated the proportion of Indian patients with EPTB with microbiologic evidence of pulmonary TB (PTB).

**METHODS:**

We characterized baseline clinical, radiological and sputum microbiologic data of 885 adult and pediatric TB patients in Chennai and Pune, India, between March 2014 and November 2018.

**RESULTS:**

Of 277 patients with EPTB, enhanced screening led to the identification of 124 (45%) with concomitant PTB, including 53 (19%) who reported a cough >2 weeks; 158 (63%) had an abnormal CXR and 51 (19%) had a positive sputum for TB. Of 70 participants with a normal CXR and without any cough, 14 (20%) had a positive sputum for TB. Overall, the incremental yield of enhanced screening of patients with EPTB to identify concomitant PTB disease was 14% (95% CI 12–16).

**CONCLUSIONS:**

A high proportion of patients classified as EPTB in India have concomitant PTB. Our results support the need for improved symptom and CXR screening, and recommends routine sputum TB microbiology screening of all Indian patients with EPTB.

## Abstract (fr)

**CONTEXTE:**

Le dépistage microbiologique des patients atteints de TB extra-pulmonaire (EPTB) pourrait éclairer des recommandations sur la prévention des aérosols et la prophylaxie pour les personnes en contact étroit avec un cas. Ces points ne sont cependant pas recommandés en routine en Inde. Nous avons estimé la proportion de patients indiens atteints d’EPTB avec documentation microbiologique de TB pulmonaire (PTB).

**MÉTHODES:**

Nous avons caractérisé les données initiales cliniques, radiologiques et microbiologiques des crachats de 885 patients adultes et pédiatriques atteints de TB à Madras et Pune, Inde, entre mars 2014 et novembre 2018.

**RÉSULTATS:**

Sur 277 patients atteints d’EPTB, le dépistage renforcé a permis d’identifier 124 (45%) patients avec PTB concomitante, dont 53 (19%) avaient rapporté une toux 2 semaines ; 158 (63%) avaient une radiographie pulmonaire (CXR) anormale et 51 (19%) avaient un examen des crachats positif pour TB. Sur 70 participants ayant une CXR normale sans toux, 14 (20%) avaient un examen des crachats positif pour TB. Dans l’ensemble, la valeur incrémentielle du dépistage renforcé des patients atteints d’EPTB visant à identifier une PTB concomitante était de 14% (IC 95% 12 16).

**CONCLUSIONS:**

Une grande proportion de patients classés comme EPTB en Inde ont une PTB concomitante. Nos résultats renforcent la nécessité d’un meilleur dépistage des symptômes et d’un meilleur dépistage par CXR. Un dépistage de routine par examen microbiologique des crachats est recommandé pour tous les patients indiens atteints d’EPTB.

TB is clinically categorized as either pulmonary TB (PTB) or extrapulmonary TB (EPTB).^1^ PTB is the most common clinical presentation of TB disease.^2^ EPTB refers to TB disease involving organs other than the lungs (e.g., pleura, lymph nodes, abdomen, genitourinary tract, skin, joints and bones, or meninges).^1^ The proportion of patients presenting with EPTB is increasing, and is reported in 10–50% of patients with TB.^3^,^4^ EPTB is more common among children, women and HIV-infected persons.^5^–^7^ TB programs classify patients as either PTB or EPTB to guide recommendations for aerosol precautions and preventive therapy for close contacts.^8^–^11^ Most TB programs, including those in India, classify patients with evidence of both extrapulmonary and pulmonary TB disease as PTB.^8^ Infection control, close contact TB prophylaxis recommendations, as well as modeling studies on community TB transmission, assume that patients with EPTB are not highly infectious.^10^,^12^,^13^ This assumption is reinforced by the difficulty in demonstrating microbiologic evidence of infection among these patients.^14^

Most TB programs recommend routine screening of patients with EPTB for pulmonary disease, only if they report symptoms suggestive of pulmonary involvement (e.g., cough >2 weeks) or have chest radiograph (CXR) findings concerning for PTB.^15^–^17^ Since 2010, the Indian National Tuberculosis Elimination Programme (NTEP) recommends routine symptom screening for all adults and children with EPTB, as well as sputum microbiology testing and a CXR for those with a reported history of cough.^18^ In 2016, the NTEP broadened its guidelines by recommending a CXR for all patients with EPTB, regardless of cough history. The revised 2016 NTEP guidelines also recommend sputum microbiology testing for such patients with any cough, significant weight loss, fever >2 weeks or presence of any CXR abnormality.^19^–^22^

It is not clear what proportion of Indian patients with EPTB have concomitant PTB disease. In low TB burden settings, there is evidence of infectiousness among 19–24% of patients with EPTB without any CXR abnormalities.^23^,^24^ However, in the absence of NTEP recommendations for routine sputum microbiology testing irrespective of symptoms or CXR abnormality, it is not clear what proportion of Indian patients with EPTB might be actually infectious. Therefore, we undertook a study in India to estimate the proportion of patients with EPTB who may be contributing to community TB transmission.

## STUDY DESIGN AND METHODS

### Patient population and study procedures

In India, patients with suspected TB are typically referred to public NTEP clinics for diagnosis and treatment. The NTEP recommends routine symptom screening of all patients with EPTB and perform a CXR, if available, followed by targeted sputum microbiologic testing for patients reporting cough or for those with an abnormal CXR finding.^21^ The NTEP guides clinicians to classify patients as EPTB or PTB, based on their clinical judgment and available clinical, radiologic and microbiologic data. Patients with EPTB are classified as EPTB only, if NTEP clinicians find no clinical, radiologic or microbiologic evidence of PTB disease.^22^ The Indian NTEP recommends that patients diagnosed with both EPTB and PTB be classified as PTB and advises close contact prophylaxis.^11^ Between March 2014 and June 2018, we enrolled newly diagnosed presumptive drug-susceptible TB patients, classified as either PTB or EPTB by the NTEP clinics in Pune and Chennai, into the prospective Cohort for TB Research with Indo-US Medical Partnership (CTRIUMPh) study, described in detail elsewhere.^25^

Patients with pre-enrollment classification of EPTB or PTB were eligible to enroll in the CTRIUMPh study within 7 days of TB treatment initiation. All consenting participants completed a standardized symptom screening, physical examination, and CXR (if not already available), and provided two sputum samples for sputum microbiology testing (acid-fast bacilli [AFB] smear, TB culture and nucleic acid amplifying testing using Xpert^®^ MTB/RIF[Cepheid, Sunnyvale, CA, USA]). Participants also consented to have additional histopathology, radiologic and microbiology results abstracted from Indian NTEP clinic records, when available.

### Radiologic finding definitions

All CXRs were digitally archived and read by two independent clinicians using the Timika scoring system, and a third independent clinician reading for consensus adjudication.^26^ All CXR readers were blinded to pre-enrollment classification of PTB or EPTB. CXRs with opacities in the lung parenchyma in any of the three zones were defined as “CXR abnormal”. Among those defined as “CXR abnormal”, those with upper zone opacities or presence of any cavity or miliary pattern were further defined as “CXR findings consistent with PTB”. CXRs with any opacity confined to middle or lower zone without any cavity or miliary pattern TB disease were labeled as “CXR abnormal, but not consistent with PTB”. CXR findings of pleural effusion or intrathoracic (mediastinal/hilar) lymphadenopathy without any opacities in the lung parenchyma were considered extrapulmonary.

### Statistical analyses

We included all TB patients enrolled in CTRIUMPh with a pre-enrollment classification of EPTB for this analysis and did not calculate a separate sample size. We used descriptive statistics to assess the patients classified by the NTEP clinics as EPTB or PTB. Categorical variables and continuous variables were compared using χ^2^ or Fisher exact test, or the Wilcoxon rank-sum test, as appropriate. We further compared the characteristics of patients with EPTB by baseline CXR and sputum TB microbiology findings, among those who did and did not report any history of cough. We also characterized sputum TB microbiology findings among those with a normal CXR and who did not report any history of cough. Factors associated with a positive sputum TB microbiology for patients with EPTB were assessed using univariate and multivariable logistic regression models. Two-sided *P* values at 0.05 alpha level were used to evaluate statistical significance. Data were analyzed using Stata Statistical Software: Release 14. (StataCorp LP, College Station, TX, USA).

### Ethical approvals

All study participants provided informed consent before enrollment into the CTRIUMPh study. The CTRIUMPh study received ethical approvals from the institutional review boards of the National Institute for Research in Tuberculosis, Chennai, India (2013003); Byramjee Jeejeebhoy Government Medical College, Pune, India (IRB00003631); and Johns Hopkins University, Baltimore, MD, USA (NA_00089784).

## RESULTS

Of the 885 newly diagnosed presumed drug-susceptible TB patients, 277 (31%) had a pre-enrollment diagnosis of EPTB only. Among these 277 patients with EPTB, 136 (49%) presented with only localized, superficial lymphadenopathy, of whom 43 (32%) were children. Other types of EPTB included pleural, abdominal and bone disease in respectively 23%, 11% and 6% (Figure). The remaining 11% presented with less common clinical manifestations, including meningitis, and skin and genitourinary disease.

**Figure:**
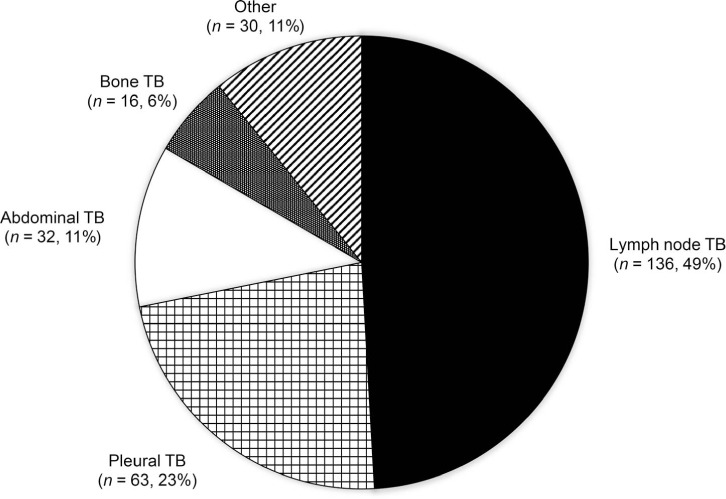
Frequency of extrapulmonary sites among patients diagnosed with extrapulmonary TB under the National Tuberculosis Elimination Programme in India.

### Characteristics of patients with EPTB

To identify concomitant PTB disease among patients with EPTB, we assessed baseline TB symptoms, CXR findings and sputum TB microbiology among all patients referred by the NTEP with a pre-enrollment classification of EPTB but without a concomitant PTB diagnosis. Of the 277 NTEP classified EPTB alone participants, 275 (99%) had baseline symptom screening data available. As shown in Table 1, 90 (33%) of them reported cough of any duration and 53 (19%) reported cough >2 weeks; 30% reported fever >2 weeks, 45% reported weight loss, and 29% reported night sweats in the past 1 month prior to their TB diagnosis. Of the 250 (90%) participants with CXR readings available, 158 (63%) had an abnormal CXR and 61 (24%) had a CXR reading consistent with PTB (i.e., apical infiltrates, cavitary disease or miliary infiltrates). Of the 268 (96%) participants with sputum microbiologic data available, 51 (19%) had at least one positive result for TB; including 11 (4%) with a positive AFB smear, 36 (14%) with a positive TB culture and 18 (8%) with a positive GeneXpert result. Overall, of the 277 patients initially classified as EPTB, 124 (45%) had clinical, radiological and/or microbiological findings consistent with concomitant PTB disease. Overall, the incremental yield of enhanced screening of patients with EPTB to identify concomitant PTB disease was 14% (95% confidence interval [CI] 12–16).

**Table 1:** Baseline characteristics of patients with EPTB and PTB in India

Characteristics	All TB patients (*n* = 885) *n* (%)	EPTB (*n* = 277) *n* (%)	PTB (*n* = 608) *n* (%)	*P* value
Age, years, median [IQR]	28 [16–42]	23 [13–34]	30 [18–45]	<0.001
Male sex	505 (57)	143 (52)	362 (60)	0.03
Ever^*^ smoked^†^	166 (27)	19 (11)	147 (32)	<0.001
Alcohol use^‡^ (AUDIT score ≥8)^*^	167 (27)	18 (11)	149 (34)	<0.001
HIV-infected^§^	67 (8)	27 (11)	40 (7)	<0.001
HbA1C, %, median [IQR]^*^	5.6 [5.1–6.1]	5.2 [5.0–5.7]	5.7 [5.1–6.3]	<0.001
Body mass index in ≥15 years, kg/m^2^, median [IQR]	18.2 [16.0–21.2]	20.0 [17.3–23.8]	17.6 [15.7–20.2]	<0.001
Any reported cough (past 30 days)	655 (74)	90 (33)	565 (93)	<0.001
Cough >2 weeks (past 30 days)	550 (62)	53 (19)	497 (82)	<0.001
Fever >2 weeks (past 30 days)	412 (47)	83 (30)	329 (54)	<0.001
Any weight loss (past 30 days)	569 (66)	120 (45)	449 (76)	<0.001
Night sweats (past 30 days)	360 (41)	80 (29)	280 (46)	<0.001
Any CXR abnormality^¶^	679 (86)	158 (63)	521 (96)	<0.001
CXR abnormality consistent with pulmonary TB^#^	497 (73)	61 (39)	436 (84)	<0.001
Any positive sputum TB microbiology (i.e., any AFB smear/Xpert/culture)	528 (61)	51 (19)	477 (79)	<0.001
Positive sputum AFB smear	392 (45)	11 (4)	381 (63)	<0.001
Positive sputum TB culture	494 (57)	36 (14)	458 (76)	<0.001
Positive sputum TB NAAT (Xpert)	399 (53)	18 (8)	381 (72)	<0.001

^*^ Excludes children and adolescents <18 years who were not administered the questionnaires or tested or missing data for smoking, alcohol use by AUDIT, HbA1c testing for diabetes or prediabetes.

^†^Self-reported current and former smoking among adults (≥18 years).

^‡^Self-reported use of alcohol among adults (≥18 years) in the past 12 months assessed using a 10-item AUDIT questionnaire at baseline.

^§^Excludes children <14 years and not born to a HIV-infected mother or with missing data.

^¶^Defined using Timika Scoring System (https://pubmed.ncbi.nlm.nih.gov/20861290/).

^#^Apical infiltrates defined as opacity in the upper zone, or presence of any cavity, or miliary pattern on CXR. No pleural effusion and mediastinal nodes in the lung and not considered for findings consistent with pulmonary TB.

EPTB = extrapulmonary TB; PTB = pulmonary TB; IQR = interquartile range; AUDIT = Alcohol Use Disorder Identification Test; HbA1c = hemoglobin A1c; CXR = chest radiograph; AFB = acid-fast bacilli; NAAT = nucleic acid amplification test.

### Positive chest X-ray or sputum without cough history

To further examine the value of routine CXR and sputum TB microbiology screening among patients with EPTB, we analyzed CXR and sputum microbiological test results among participants with and without any reported history of cough (Table 2). Among 185 participants without any reported history of cough, 166 had a CXR result available; 93 (56%) had an abnormal CXR, including 33 (20%) with a CXR consistent with PTB disease. Among 90 participants who reported a history of cough, 83 had a CXR result available; 64 (77%) had an abnormal CXR, including 28 (34%) with a CXR consistent with PTB disease—a significantly higher proportion than participants without cough history. Among the 185 participants without any reported history of cough, 180 had sputum microbiologic data available; 32 (18%) had at least one positive result for TB, including 7 (4%) with a positive AFB smear, 21 (12%) with a positive TB culture, and 11 (7%) with a positive GeneXpert result. Among the 90 participants who reported a history of cough, 86 had sputum microbiologic data available; 19 (22%) participants had at least one positive sputum test result for TB, including 4 (5%) with a positive AFB smear, 15 (17%) with a positive TB culture, and 7 (8%) with a positive GeneXpert result. There was no significant difference in positive sputum TB microbiology results between participants with and without cough history.

**Table 2:** Baseline CXR and sputum microbiology findings among patients with EPTB without cough in India

Findings of pulmonary involvement	No cough (*n* = 185) *n* (%)	Any cough (*n* = 90) *n* (%)	*P* value^*^	Cough <2 weeks (*n* = 37) *n* (%)	Cough >2 weeks (*n* = 53) *n* (%)	*P* value^†^
CXR abnormality			0.001			0.31
Abnormal CXR^‡^	93 (56)	64 (77)		25 (71)	39 (81)	
Normal CXR	73 (44)	19 (23)		10 (29)	9 (19)	
Not available	19	7		2	5	
CXR abnormality consistent with pulmonary TB			0.02			0.002
CXR consistent with pulmonary TB^§^	33 (20)	28 (34)		5 (14)	23 (48)	
CXR not consistent with pulmonary TB^¶^	133 (80)	55 (66)		30 (86)	25 (52)	
Not available	19	7		2	5	
Any sputum TB microbiology test result (i.e., any AFB smear/Xpert/culture)			>0.95			0.28
Positive	32 (18)	19 (22)		5 (15)	14 (27)	
Negative	148 (83)	67 (78)		29 (85)	38 (73)	
Not available	5	4		3	1	
Sputum AFB smear result			0.25			>0.95
Positive	7 (4)	4 (5)		1 (3)	3 (6)	
Negative	171 (96)	82 (95)		33 (97)	49 (94)	
Not available	7	4		3	1	
Sputum TB culture result			0.47			0.38
Positive	21 (12)	15 (18)		4 (12)	11 (22)	
Negative	157 (88)	70 (82)		30 (88)	40 (78)	
Not available	7	5		3	2	
Sputum TB NAAT result (Xpert)			>0.95			0.04
Positive	11 (7)	7 (9)		0	7 (15)	
Negative	140 (93)	70 (91)		31 (100)	39 (85)	
Not available	34	13		6	7	

^*^ Compares patients with EPTB without any reported cough in the past 30 days to those with reported any cough.

^†^Among those with a reported cough, compares patients with EPTB with a reported cough <2 weeks to those with a reported cough of >2 weeks.

^‡^Defined using Timika Scoring System (https://pubmed.ncbi.nlm.nih.gov/20861290/).

^§^With apical infiltrates defined as opacity in the upper zone, or presence of any cavity, or miliary pattern; no pleural effusion or mediastinal nodes observed in the lung and not considered as findings consistent with pulmonary TB.

^¶^Includes those with a normal CXR as well.

CXR = chest radiograph; EPTB = extrapulmonary TB; AFB = acid-fast bacilli; NAAT = nucleic acid amplification test.

### Positive sputum without cough and with a normal chest X-ray

To further evaluate the value of routine sputum microbiologic testing among all patients with EPTB, we assessed sputum test results among a subset of 73 patients who did not report any history of cough and also had a normal CXR reading (Table 3). Of 70 (96%) participants with sputum microbiologic data available, 14 (20%) had microbiologic evidence of PTB disease, including 4 (6%) with a positive AFB smear, 10 (14%) with a positive TB culture, and 1 (2%) with a positive GeneXpert result.

**Table 3:** Baseline sputum microbiology among Indian patients with EPTB without cough history and normal CXR findings

Sputum microbiology for TB test results	No cough + normal CXR^*^ (*n* = 73) *n* (%)
Any sputum TB microbiology test result (i.e., any AFB smear/Xpert/culture)	
Positive	14 (20)
Negative	56 (80)
Not available	3
Sputum AFB smear result	
Positive	4 (6)
Negative	66 (94)
Not available	3
Sputum TB culture result	
Positive	10 (14)
Negative	60 (86)
Not available	3
Sputum TB NAAT result (Xpert)	
Positive	1 (2)
Negative	52 (98)
Not available	20

^*^ Pleural effusion or intrathoracic (mediastinal or hilar) nodes without opacity in the lung parenchyma were considered normal CXR. Abnormal CXR scoring were defined using the Timika scoring system (https://pubmed.ncbi.nlm.nih.gov/20861290/).

EPTB = extrapulmonary TB; CXR = chest radiograph; AFB = acid-fast bacilli; NAAT = nucleic acid amplification test.

### Risk factors for positive sputum

To determine whether a subset of patients with EPTB were more likely to have microbiologic evidence of concomitant PTB disease, we assessed risk factors associated with a positive sputum microbiology for TB. We did not find any risk factor to be independently associated with a positive sputum microbiology for TB among the 268 patients with sputum microbiologic data available (data not shown).

## DISCUSSION

Based on WHO recommendations, TB programs classify patients with evidence of both extrapulmonary and pulmonary TB disease as PTB to guide recommendations for respiratory precautions and preventive therapy for close contacts.^8^–^10^ This is based on the assumption that patients with EPTB are less infectious than those with PTB disease.^27^ In addition, TB transmission modeling studies also assume that patients with EPTB are not highly infectious to others in the community.^13^,^28^ This is under the assumption that all patients diagnosed as EPTB are effectively screened and ruled out for concomitant PTB disease, and only have extrapulmonary disease.

With 26% of the global burden of TB disease, India recommends routine symptom screening and a CXR for all patients with EPTB.^19^,^29^ Acknowledging challenges with CXR availability, as well as lack of standardization in CXR reading and reporting, studies assessing the implementation of national guidelines for screening patients with EPTB for concomitant PTB by symptoms and CXR in high TB burden settings, including India, are sparse.^30^–^33^ In our analyses, 45% of patients initially classified as EPTB by the Indian NTEP, actually had clinical, radiological and/or microbiological findings consistent with concomitant PTB disease. Although the Indian national guidelines recommend symptom and CXR screening of patients with EPTB, and reclassification of patients with reported cough >2 weeks or with CXR abnormality as PTB, one-third of patients with EPTB interviewed in our study reported a history cough >2 weeks, 63% had an abnormal CXR and 24% had a CXR reading consistent with PTB. These findings raise concerns that many patients in India with both EPTB and concomitant PTB disease may be misclassified as EPTB alone. Additionally, our findings support the need for future studies to assess the programmatic implementation of the national screening guidelines among patients diagnosed as EPTB in high TB burden settings, including India.

Furthermore, our study demonstrated that 19% of patients initially classified as EPTB by the program also had concomitant sputum microbiologic evidence for PTB, 20% of whom denied any history of cough and also had a normal CXR reading. Two prior studies from low TB burden settings demonstrated that 19% (Saudi Arabia) and 24% (United States) of patients with EPTB and a normal CXR reading had a positive sputum microbiology for TB.^23^,^24^ To our knowledge, our study is the first to confirm the high risk of concomitant PTB disease among patients with EPTB irrespective of their cough symptoms or CXR abnormality in a high TB burden setting. Our findings support the recommendation for routine sputum microbiology screening for all patients with EPTB to identify concomitant PTB disease, as missing a diagnosis of PTB has implications for potential community transmission in high TB burden settings, including India.

Our study had several potential limitations. First, of all patients evaluated for suspected EPTB at our recruitment clinics, only 45% were enrolled and we did not keep track of all patients initiated on TB treatment; our study participants may therefore not be representative of all patients diagnosed with EPTB in India’s NTEP. Second, symptom, CXR and sputum microbiology data were missing for some of our study participants, including 16% of whom did not have sputum GeneXpert testing results and 10% who did not have CXR data available. We therefore may have underestimated the number of patients with EPTB with or without concomitant pulmonary involvement.

Despite these limitations, our findings have several programmatic implications for the NTEP in India and other similar high TB burden settings. Since patients with EPTB without evidence of concomitant pulmonary disease are typically assumed to be not highly infectious, misclassification of patients with EPTB has implications for the education of patients and their close contacts about the need for respiratory precautions and preventive therapy for TB. Our data support the 2017 INDEX-TB recommendation of routine symptom screening and CXR for all patients with EPTB. It should be noted that our study also highlights the need to consider routine sputum microbiology evaluation for all patients with EPTB, regardless of their reported symptoms or CXR findings, as we demonstrated an incremental yield of 14% additional PTB cases by enhanced screening.

According to the WHO, contact investigation is typically not recommended for index EPTB cases, in the assumption that they are free of concomitant PTB disease and low in infectiousness.^10^ However, given that nearly a fifth of patients with EPTB are sputum microbiologically confirmed and eligible for contact evaluation as per the Indian NTEP guidelines, our study findings highlight the critical need to screen all close contacts of patients with EPTB for TB.^21^ Finally, our study also has implications for studies that model community TB transmission, as many of these models do not account for the proportion of patients with EPTB, who are misclassified as not having PTB, and who could be contributing to community TB transmission.

## CONCLUSION

Our results highlight the need for additional training and education of Indian healthcare providers about the need for improved symptom and CXR screening of all patients with EPTB, its implications on reclassification of EPTB as PTB and close-contact prophylaxis. Our study also supports the recommendation for routine sputum TB microbiology of all Indian patients with EPTB, regardless of clinical presentation or CXR findings.
